# Supplementary material for: Finding common ground: Understanding and engaging with science mistrust in the Great barrier reef region
Source: PLoS One. 2024 Aug 16;19(8):e0308252. doi: 10.1371/journal.pone.0308252 (PMC11329155; doi:10.1371/journal.pone.0308252)
Supplement: S1 Table — (DOCX) [file pone.0308252.s001.docx]

**S1 Table.** **Results of ordinal regression models testing the relationship between survey respondents’ *’trust* [in] *the science about waterway health and management’* and predictor variables from five survey questions about *respondent demography.*** Demographic variables include gender, age, years living in the region, work sector and proportion of household income from waterways-related business or employment. Cumulative odds ratios indicate the predicted likelihood of increased or decreased *trust in science* corresponding to higher ratings in the predictor variable (values greater than one represent an increased likelihood while values less than one suggest decreased likelihoods). Variables with significant (p < 0.05) effects are indicated in bold font.

| Survey question & response options | Short variable name | Model results | | | |
| --- | --- | --- | --- | --- | --- |
|  |  | **Regression coefficient**  **(log odds)** | **Cumulative odds ratio** | **Z value** | **p value** |
| *How do you describe your gender?* Binary scale (1=female, 2=male)  Note: due to low numbers, “other/non-binary” and “prefer not to say” response options were excluded from the analysis. | **Gender** | **-0.343** | **0.71** | **-4.070** | **0.000** |
| *What is your age?*  7 categories in scale (1=18-24, 2=25-34, 3=35-34, 4=45-54, 5=55-64, 6=65-74, 7= 75+) | Age | 0.019 | 1.02 | -0.665 | 0.371 |
| *For how many years have you lived in the region?* | **Duration of residence** | **-0.014** | **0.99** | **-5.579** | **0.001** |
| *What sector do you mainly work in?*  Response options included 22 employment sectors (including ‘None’ and ‘Other Services’) corresponding to Australian Bureau of Statistics Census categories. Sectors were grouped into four major types: (i) *Primary industries* included ‘Agricultural’, ‘Forestry’, ‘Fishing’, and ‘Mining’; (ii) *Professional & technical services* included ‘Information Media and Telecommunications’, ‘Financial and Insurance Services’, ‘Professional, Scientific and Technical Services’, ‘Education and Training’, and ‘Health Care and Social Assistance’; (iii) *Skilled trades* included ‘Manufacturing’, ‘Electricity, Gas, Water and Waste Services’, and ‘Construction’; (iv) *Urban services* included ‘Wholesale Trade’, ‘Retail Trade’, Accommodation and Food Services’, ‘Transport, Postal and Warehousing’, ‘Rental, Hiring and Real Estate Services’, ‘Administrative and Support Services’, ‘Public Administration and Safety’, and ‘Arts and Recreation Services’. | **Primary industries** | **-0.477** | **0.62** | **-3.492** | **0.002** |
|  | **Professional & technical services** | **0.327** | **1.39** | **3.169** | **0.001** |
|  | Skilled trades | -0.252 | 0.78 | -1.362 | 0.186 |
|  | Urban services | 0.012 | 1.01 | 0.106 | 0.831 |
| *What proportion of your household income is from waterways-related businesses or employment?*  5 categories in scale (1=None, 2=a small amount (less than 25%), 3=a moderate amount (around 50%), 4=a large amount (around 75%), 5=all my household income (100%)) | **Income** | **0.085** | **1.09** | **2.265** | **0.023** |
