# Supplementary material for: Finding common ground: Understanding and engaging with science mistrust in the Great barrier reef region
Source: PLoS One. 2024 Aug 16;19(8):e0308252. doi: 10.1371/journal.pone.0308252 (PMC11329155; doi:10.1371/journal.pone.0308252)
Supplement: S2 Table — (DOCX) [file pone.0308252.s002.docx]

**S2 Table.** **Results of ordinal regression models testing the relationship between survey respondents’ *’trust* [in] *the science about waterway health and management’* and predictor variables from survey questions about *values attributed to regional waterways*, and mean rating scores (±SE) from four groups with differing stated *trust in science* (strongly sceptical, mildly sceptical, mildly trusting, strongly trusting) for each predictor variable**. Cumulative odds ratios indicate the predicted likelihood of increased or decreased *trust in science* corresponding to higher ratings in the predictor variable (values greater than one represent an increased likelihood while values less than one suggest decreased likelihoods). Variables with significant (p < 0.05) effects are indicated in bold font.

| Survey question and response options | Question items | Short variable name | Model results | | | | Mean rating scores (±SE) from four groups with differing stated trust in science | | | | | | | |
| --- | --- | --- | --- | --- | --- | --- | --- | --- | --- | --- | --- | --- | --- | --- |
|  |  |  |  |  |  |  | **Strong Sceptic** | | **Mild Sceptic** | | **Mild Trust** | | **Strong Trust** | |
|  |  |  | **Regression coefficient**  **(log odds)** | **Cumulative odds ratio** | **Z value** | **p value** | **Mean** | **SE** | **Mean** | **SE** | **Mean** | **SE** | **Mean** | **SE** |
| Waterway values  *“How much do you value the following aspects of waterways in the region?”*  10-point scale (1=I don’t value this at all, 10=I value this extremely highly) | The waterways have rich heritage to First Nations people | **First Nations Heritage** | **0.106** | **1.11** | **4.521** | **0.000** | **5.56** | 0.279 | **6.48** | 0.128 | **7.13** | 0.081 | **8.14** | 0.105 |
|  | The waterways offer a place for local residents to enjoy recreation activities | **Local recreation** | **0.096** | **1.11** | **2.944** | **0.003** | **8.20** | 0.186 | **8.17** | 0.097 | **8.51** | 0.055 | **9.24** | 0.063 |
|  | The fact that the waterways exist, even if I don’t use or directly benefit from them | **Existence value** | **0.094** | **1.09** | **2.758** | **0.006** | **8.27** | 0.171 | **8.43** | 0.085 | **8.65** | 0.056 | **9.46** | 0.054 |
|  | The waterways are an important attraction for tourists visiting the region | **Tourism attraction** | **0.079** | **1.08** | **3.414** | **0.001** | **6.48** | 0.269 | **7.05** | 0.135 | **7.63** | 0.079 | **8.53** | 0.099 |
|  | Our waterways are recognised nationally and internationally for their iconic status (e.g. World Heritage, RAMSAR sites). | **Iconic status** | **0.050** | **1.05** | **2.104** | **0.035** | **7.19** | 0.246 | **7.47** | 0.115 | **7.93** | 0.070 | **8.42** | 0.105 |
|  | The waterways support a variety of native habitats, plants and wildlife | Biodiversity | 0.024 | 1.02 | 1.010 | 0.313 | 8.16 | 0.212 | 8.02 | 0.111 | 8.38 | 0.067 | 9.05 | 0.080 |
|  | The waterways support commercial fishing | Commercial fishing | 0.020 | 1.02 | 0.923 | 0.356 | 6.41 | 0.258 | 6.68 | 0.129 | 7.19 | 0.078 | 7.26 | 0.131 |
|  | The waterways provide a place where people can pass down knowledge, traditions, and a way of life | Generational knowledge | 0.012 | 1.02 | 0.414 | 0.679 | 7.32 | 0.236 | 7.73 | 0.108 | 8.09 | 0.066 | 8.70 | 0.091 |
|  | The waterways support our local economy | Local economic value | -0.041 | 0.97 | -1.365 | 0.172 | 7.75 | 0.202 | 7.84 | 0.101 | 8.06 | 0.062 | 8.58 | 0.087 |
|  | The waterways support recreational fishing | Recreational fishing | -0.041 | 0.96 | -1.543 | 0.123 | 7.73 | 0.225 | 7.59 | 0.114 | 7.96 | 0.066 | 8.28 | 0.108 |
|  | The waterways support local agriculture | **Local agriculture** | **-0.061** | **0.93** | **-2.818** | **0.005** | **6.99** | 0.273 | **6.89** | 0.124 | **7.16** | 0.079 | **7.28** | 0.129 |
|  | The waterways support local aquaculture | Local aquaculture | -0.005 | 0.99 | -0.310 | 0.757 | 5.58 | 0.270 | 5.66 | 0.145 | 6.04 | 0.100 | 6.10 | 0.146 |
|  | The waterways support mining, ports and shipping in our region | **Mining & shipping** | **0.045** | **1.05** | **2.234** | **0.026** | **5.84** | 0.274 | **6.17** | 0.131 | **6.79** | 0.083 | **7.03** | 0.139 |
|  | The waterways provide a place where people can study and learn about the natural environment | Environmental knowledge | 0.026 | 1.02 | 0.837 | 0.403 | 5.58 | 0.270 | 5.66 | 0.145 | 6.04 | 0.100 | 6.10 | 0.146 |
