# Supplementary material for: Finding common ground: Understanding and engaging with science mistrust in the Great barrier reef region
Source: PLoS One. 2024 Aug 16;19(8):e0308252. doi: 10.1371/journal.pone.0308252 (PMC11329155; doi:10.1371/journal.pone.0308252)
Supplement: S3 Table — (DOCX) [file pone.0308252.s003.docx]

**S3 Table.** **Results of ordinal regression models testing the relationship between survey respondents *’trust* [in] *the science about waterway health and management’* and predictor variables from survey questions about *perceptions of waterway governance*, and mean rating scores (±SE) from four groups with differing stated *trust in science* (strongly sceptical, mildly sceptical, mildly trusting, strongly trusting) for each predictor variable**. Cumulative odds ratios indicate the predicted likelihood of increased or decreased *trust in science* corresponding to higher ratings in the predictor variable (values greater than one represent an increased likelihood while values less than one suggest decreased likelihoods). Variables with significant (p < 0.05) effects are indicated in bold font.

| Survey question and response options | Question items | Short variable name | Model results | | | | Mean rating scores (±SE) from four groups with differing stated trust in science | | | | | | | |
| --- | --- | --- | --- | --- | --- | --- | --- | --- | --- | --- | --- | --- | --- | --- |
|  |  |  |  |  |  |  | **Strong Sceptic** | | **Mild Sceptic** | | **Mild Trust** | | **Strong Trust** | |
|  |  |  | **Regression coefficient** | **Cumulative odds ratio** | **Z value** | **p value** | **Mean** | **SE** | **Mean** | **SE** | **Mean** | **SE** | **Mean** | **SE** |
| Governance perceptions:  *“Please rate your level of agreement with the following statements.”*  10-point scale (1=Very Strongly Disagree, 10=Very Strongly Agree)​ | I think that decisions about managing local waterways are made in a fair way | **Management decisions are fair** | **0.320** | **1.38** | **10.495** | **0.000** | **3.65** | 0.235 | **4.88** | 0.098 | **5.77** | 0.063 | **6.23** | 0.116 |
|  | I feel able to have input into the management of waterways in my region if I choose to | **Input into management of waterways** | **0.176** | **1.19** | **7.207** | **0.000** | **2.91** | 0.204 | **4.15** | 0.095 | **4.95** | 0.072 | **5.91** | 0.126 |
|  | I think that tourism uses of waterways in our region are well managed | **Tourism uses well managed** | **0.142** | **1.15** | **5.002** | **0.000** | **5.50** | 0.227 | **5.47** | 0.097 | **6.33** | 0.057 | **7.00** | 0.101 |
|  | I think that the fisheries in our region are well managed | **Fisheries well managed** | **0.090** | **1.09** | **3.076** | **0.002** | **4.74** | 0.249 | **4.93** | 0.090 | **5.90** | 0.063 | **6.42** | 0.110 |
|  | Overall, I feel satisfied with how local waterways are managed | Satisfied with management | 0.040 | 1.04 | 1.434 | 0.152 | 3.65 | 0.235 | 4.88 | 0.098 | 5.77 | 0.063 | 6.23 | 0.116 |
|  | I feel I personally have some influence over how local waterways are managed | Personal influence on waterway management | -0.015 | 0.99 | -0.641 | 0.522 | 2.60 | 0.193 | 3.73 | 0.095 | 4.29 | 0.073 | 4.81 | 0.130 |
|  | I think that ports and shipping in our region are well managed | Ports & shipping well managed | -0.045 | 0.96 | -1.631 | 0.103 | 5.24 | 0.233 | 5.36 | 0.094 | 5.92 | 0.064 | 6.21 | 0.116 |
|  | I ***do not*** have fair access to all the waterways in my region that I would like to use | **Don't have fair access to waterways** | **-0.082** | **0.92** | **-4.818** | **0.000** | **5.16** | 0.281 | **4.76** | 0.115 | **4.61** | 0.081 | **3.91** | 0.129 |
|  | I think that agricultural uses of waterways in our region are well managed | **Agricultural uses well managed** | **-0.223** | **0.80** | **-8.215** | **0.000** | **5.44** | 0.286 | **5.15** | 0.102 | **5.68** | 0.069 | **5.86** | 0.122 |
|  | I think that aquaculture uses of waterways in our region are well managed | Aquaculture uses well managed | 0.050 | 1.05 | 1.474 | 0.140 | 4.88 | 0.226 | 5.11 | 0.080 | 5.93 | 0.056 | 6.28 | 0.105 |
