# Supplementary material for: Finding common ground: Understanding and engaging with science mistrust in the Great barrier reef region
Source: PLoS One. 2024 Aug 16;19(8):e0308252. doi: 10.1371/journal.pone.0308252 (PMC11329155; doi:10.1371/journal.pone.0308252)
Supplement: S4 Table — (DOCX) [file pone.0308252.s004.docx]

**S4 Table.** **Results of ordinal regression models testing the relationship between survey respondents *’trust* [in] *the science about waterway health and management’* and predictor variables from survey questions about *recreational uses of regional waterways*, and mean rating scores (±SE) from four groups with differing stated *trust in science* (strongly sceptical, mildly sceptical, mildly trusting, strongly trusting) for each predictor variable**. Cumulative odds ratios indicate the predicted likelihood of increased or decreased *trust in science* corresponding to higher ratings in the predictor variable (values greater than one represent an increased likelihood while values less than one suggest decreased likelihoods). Variables with significant (p < 0.05) effects are indicated in bold font.

| Survey question and response options | Question items | Short variable name | Model results | | | | Mean rating scores (±SE) from four groups with differing stated trust in science | | | | | | | |
| --- | --- | --- | --- | --- | --- | --- | --- | --- | --- | --- | --- | --- | --- | --- |
|  |  |  |  |  |  |  | **Strong Sceptic** | | **Mild Sceptic** | | **Mild Trust** | | **Strong Trust** | |
|  |  |  | **Regression coefficient**  **(log odds)** | **Cumulative odds ratio** | **Z value** | **p value** | **Mean** | **SE** | **Mean** | **SE** | **Mean** | **SE** | **Mean** | **SE** |
| Recreation uses of regional waterways:  *“When visiting all the different waterways in the region, in the past 12 months, what recreational activities have you participated in? Select all that apply.”*  Response scale: 0 = No, 1 = Yes | Wildlife watching and appreciating nature | **Appreciating nature** | **0.425** | **1.53** | **4.864** | **0.000** | **0.404** | 0.042 | **0.425** | 0.024 | **0.466** | 0.017 | **0.632** | 0.023 |
|  | Snorkelling/freediving/Scuba diving | **Snorkelling & diving** | **0.248** | **1.28** | **2.280** | **0.023** | **0.206** | 0.034 | **0.192** | 0.019 | **0.247** | 0.015 | **0.305** | 0.022 |
|  | Swimming | **Swimming** | **0.207** | **1.23** | **2.186** | **0.029** | **0.411** | 0.042 | **0.511** | 0.024 | **0.453** | 0.017 | **0.423** | 0.023 |
|  | Picnics & barbecues | Picnics & barbecues | 0.149 | 1.16 | 1.702 | 0.089 | 0.383 | 0.041 | 0.477 | 0.024 | 0.526 | 0.017 | 0.571 | 0.023 |
|  | Wind-powered water sports (e.g. kite surfing) | Wind-powered watersports | 0.119 | 1.13 | 0.358 | 0.720 | 0.028 | 0.014 | 0.007 | 0.004 | 0.018 | 0.005 | 0.015 | 0.006 |
|  | Paddling/canoeing/kayaking | Canoeing & kayaking | -0.010 | 0.99 | -0.097 | 0.923 | 0.170 | 0.032 | 0.196 | 0.019 | 0.211 | 0.014 | 0.244 | 0.020 |
|  | Camping | Camping | -0.039 | 0.96 | -0.399 | 0.690 | 0.305 | 0.039 | 0.295 | 0.022 | 0.328 | 0.016 | 0.327 | 0.022 |
|  | Boating or sailing | Boating or sailing | -0.059 | 0.94 | -0.576 | 0.565 | 0.362 | 0.041 | 0.267 | 0.021 | 0.294 | 0.016 | 0.296 | 0.021 |
|  | Fishing (e.g. line, spear, crabbing/yabbies) | **Fishing** | **-0.196** | **0.82** | **-2.182** | **0.029** | **0.589** | 0.042 | **0.466** | 0.024 | **0.453** | 0.017 | **0.423** | 0.023 |
|  | Motor-powered water sports (e.g. water skiing, jet ski) | **Motorised watersports** | **-0.397** | **0.67** | **-2.634** | **0.008** | **0.085** | 0.024 | **0.121** | 0.016 | **0.079** | 0.009 | **0.065** | 0.012 |
|  | Exercise | **Exercise** | **0.314** | **1.37** | **3.623** | **0.000** | **0.270** | 0.038 | **0.418** | 0.024 | **0.492** | 0.017 | **0.538** | 0.023 |
