# Supplementary material for: Finding common ground: Understanding and engaging with science mistrust in the Great barrier reef region
Source: PLoS One. 2024 Aug 16;19(8):e0308252. doi: 10.1371/journal.pone.0308252 (PMC11329155; doi:10.1371/journal.pone.0308252)
Supplement: S5 Table — (DOCX) [file pone.0308252.s005.docx]

**S5 Table.** **Results of ordinal regression models testing the relationship between survey respondents *’trust* [in] *the science about waterway health and management’* and predictor variables from survey questions about *personal benefits derived from regional waterways*, and mean rating scores (±SE) from four groups with differing stated *trust in science* (strongly sceptical, mildly sceptical, mildly trusting, strongly trusting) for each predictor variable**. Cumulative odds ratios indicate the predicted likelihood of increased or decreased *trust in science* corresponding to higher ratings in the predictor variable (values greater than one represent an increased likelihood while values less than one suggest decreased likelihoods). Variables with significant (p < 0.05) effects are indicated in bold font.

| Survey question and response options | Question items | Short variable name | Model results | | | | Mean rating scores (±SE) from four groups with differing stated trust in science | | | | | | | |
| --- | --- | --- | --- | --- | --- | --- | --- | --- | --- | --- | --- | --- | --- | --- |
|  |  |  |  |  |  |  | **Strong Sceptic** | | **Mild Sceptic** | | **Mild Trust** | | **Strong Trust** | |
|  |  |  | **Regression coefficient**  **(log odds)** | **Cumulative odds ratio** | **Z value** | **p value** | **Mean** | **SE** | **Mean** | **SE** | **Mean** | **SE** | **Mean** | **SE** |
| Personal benefits derived from regional waterways:  *“Thinking about your visits and uses of waterways in the region in general, please rate your level of agreement with the following statements.”*  10-point response scale (1=Very Strongly Disagree, 10=Very Strongly Agree)​ | The waterways are important for allowing me to experience, appreciate and interact with the natural environment | **Experiencing nature** | **0.104** | **1.11** | **3.074** | **0.002** | **7.75** | 0.214 | **7.92** | 0.110 | **8.23** | 0.067 | **9.11** | 0.081 |
|  | Waterways in the region provide an important place for me to spend time with family and friends | Social opportunities | 0.054 | 1.06 | 1.729 | 0.084 | 7.65 | 0.222 | 7.58 | 0.115 | 8.00 | 0.071 | 8.77 | 0.086 |
|  | The waterways are an important source of my water supply for drinking and household use | **Domestic water supply** | **0.054** | **1.06** | **3.225** | **0.001** | **6.82** | 0.277 | **7.25** | 0.135 | **7.63** | 0.089 | **8.14** | 0.126 |
|  | Waterways in the region support my lifestyle and recreational interests | Lifestyle & recreation | 0.033 | 1.03 | 1.032 | 0.302 | 7.62 | 0.222 | 7.41 | 0.119 | 7.86 | 0.073 | 8.64 | 0.093 |
|  | I am proud of the local waterways in my region | Sense of pride | 0.007 | 1.01 | 0.277 | 0.782 | 7.24 | 0.241 | 7.40 | 0.113 | 7.72 | 0.074 | 8.22 | 0.104 |
|  | Waterways in the region are an important part of my culture | Important part of culture | -0.018 | 0.98 | -0.993 | 0.321 | 5.82 | 0.288 | 5.77 | 0.136 | 6.07 | 0.088 | 6.55 | 0.132 |
|  | Visiting waterways in the region is important for my quality of life and wellbeing | Quality of life & wellbeing | -0.039 | 0.96 | -1.093 | 0.274 | 7.51 | 0.228 | 7.61 | 0.117 | 7.95 | 0.071 | 8.74 | 0.093 |
|  | The natural beauty of waterways in the region is outstanding | Aesthetic appreciation | -0.040 | 0.96 | -1.700 | 0.089 | 7.57 | 0.228 | 7.33 | 0.127 | 7.67 | 0.080 | 8.21 | 0.111 |
|  | Waterways in the region are important for providing fresh fish and seafood for me to eat | **Providing fish & seafood** | **-0.051** | **0.95** | **-3.141** | **0.002** | **6.55** | 0.266 | **6.63** | 0.136 | **6.65** | 0.095 | **6.85** | 0.141 |
|  | If the health of waterways in my region declined, I would be personally affected | **Personal affect** | **0.151** | **1.16** | **5.519** | **0.000** | **6.97** | 0.253 | **7.24** | 0.116 | **7.68** | 0.072 | **8.57** | 0.092 |
|  | The waterways in my region are an important part of why I choose to live here | Choice of living location | 0.011 | 1.01 | 0.420 | 0.675 | 7.01 | 0.261 | 7.10 | 0.121 | 7.50 | 0.076 | 8.26 | 0.104 |
