# Supplementary material for: Finding common ground: Understanding and engaging with science mistrust in the Great barrier reef region
Source: PLoS One. 2024 Aug 16;19(8):e0308252. doi: 10.1371/journal.pone.0308252 (PMC11329155; doi:10.1371/journal.pone.0308252)
Supplement: S6 Table — (DOCX) [file pone.0308252.s006.docx]

**S6 Table.** **Results of ordinal regression models testing the relationship between survey respondents *’trust* [in] *the science about waterway health and management’* and predictor variables from survey questions about *respondents’ participation in waterway stewardship actions*, and mean rating scores (±SE) from four groups with differing stated *trust in science* (strongly sceptical, mildly sceptical, mildly trusting, strongly trusting) for each predictor variable**. Cumulative odds ratios indicate the predicted likelihood of increased or decreased *trust in science* corresponding to higher ratings in the predictor variable (values greater than one represent an increased likelihood while values less than one suggest decreased likelihoods). Variables with significant (p < 0.05) effects are indicated in bold font.

| Survey question and response options | Question items | Short variable name | Model results | | | | Mean rating scores (±SE) from four groups with differing stated trust in science | | | | | | | |
| --- | --- | --- | --- | --- | --- | --- | --- | --- | --- | --- | --- | --- | --- | --- |
|  |  |  |  |  |  |  | **Strong Sceptic** | | **Mild Sceptic** | | **Mild Trust** | | **Strong Trust** | |
|  |  |  | **Regression coefficient (log odds)** | **Cumulative odds ratio** | **Z value** | **p value** | **Mean** | **SE** | **Mean** | **SE** | **Mean** | **SE** | **Mean** | **SE** |
| Stewardship actions:  *“For the following questions, we would like to ask you about several personal actions that are intended to improve waterway health. Which of the following do you personally do?”*  Response scale: 0 = No, 1 = Yes | Contribute to environmental monitoring programs (e.g. by participating in data collection, or reporting wildlife sightings) | **Contribute to environmental monitoring** | **0.383** | **1.47** | **3.186** | **0.001** | **0.270** | 0.038 | **0.231** | 0.020 | **0.240** | 0.015 | **0.388** | 0.023 |
|  | Dispose of food scraps and rubbish appropriately (i.e. ashore, in a rubbish bin provided by council, or at home) | **Dispose of rubbish & food scraps** | **0.377** | **1.46** | **2.612** | **0.009** | 0.894 | 0.026 | 0.822 | 0.018 | 0.847 | 0.013 | 0.882 | 0.015 |
|  | Participate in local environmental restoration (e.g. weed removal, tree planting, coral restoration) | **Participate in restoration** | **0.396** | **1.49** | **3.158** | **0.002** | 0.199 | 0.034 | 0.199 | 0.019 | 0.188 | 0.014 | 0.316 | 0.022 |
|  | Participate in local environmental clean-ups (e.g. picking up rubbish, or marine debris) | Participate in cleanups | 0.067 | 1.06 | 0.650 | 0.515 | 0.489 | 0.042 | 0.420 | 0.024 | 0.431 | 0.034 | 0.553 | 0.046 |
|  | Report invasive or pest species to relevant authorities (e.g. weeds, feral animals; to council, or Biosecurity QLD) | Report invasive or pest species | -0.215 | 0.81 | -1.799 | 0.516 | 0.454 | 0.042 | 0.352 | 0.023 | 0.308 | 0.016 | 0.416 | 0.023 |
|  | Report suspicious activity to relevant authorities (e.g. illegal dumping, illegal fishing practices, chemical or oil spills) | Report suspicious activities | -0.007 | 0.99 | -0.069 | 0.035 | 0.945 | 0.041 | **0.459** | 0.024 | **0.479** | 0.017 | **0.562** | 0.023 |
|  | For people who have or use a boat... Responsible anchoring (e.g. by using a geo-spot electric motor, or anchoring only in sandy areas away from corals/seagrass) | Responsible anchoring | -0.153 | 0.86 | -1.443 | 0.149 | 0.390 | 0.041 | 0.267 | 0.021 | 0.274 | 0.015 | 0.285 | 0.021 |
|  | For people who go fishing… Responsible fishing (e.g. take only the fish you intend to eat; quickly release undersize or unidentified fish; using barbless or circle hooks) | Responsible fishing | -0.141 | 0.87 | -1.378 | 0.168 | 0.681 | 0.039 | 0.543 | 0.024 | 0.544 | 0.017 | 0.549 | 0.023 |
|  | Responsible four-wheel driving (e.g. avoiding river banks to minimise erosion, sand dunes during turtle nesting season) | Responsible four-wheel driving | -0.070 | 0.93 | -0.738 | 0.461 | 0.518 | 0.042 | 0.377 | 0.023 | 0.358 | 0.017 | 0.405 | 0.023 |
