# Supplementary material for: Finding common ground: Understanding and engaging with science mistrust in the Great barrier reef region
Source: PLoS One. 2024 Aug 16;19(8):e0308252. doi: 10.1371/journal.pone.0308252 (PMC11329155; doi:10.1371/journal.pone.0308252)
Supplement: S7 Table — (DOCX) [file pone.0308252.s007.docx]

**S7 Table.** **Results of ordinal regression models testing the relationship between survey respondents *’trust* [in] *the science about waterway health and management’* and predictor variables from survey questions about *respondents’ motivation and capacity to participate in stewardship actions to improve the health of their region’s waterways (i.e. stewardship enablers*), and mean rating scores (±SE) from four groups with differing stated *trust in science* (strongly sceptical, mildly sceptical, mildly trusting, strongly trusting) for each predictor variable**. Cumulative odds ratios indicate the predicted likelihood of increased or decreased *trust in science* corresponding to higher ratings in the predictor variable (values greater than one represent an increased likelihood while values less than one suggest decreased likelihoods). Variables with significant (p < 0.05) effects are indicated in bold font.

| Survey question and response options | Question items | Short variable name | Model results | | | | Mean rating scores (±SE) from four groups with differing stated trust in science | | | | | | | |
| --- | --- | --- | --- | --- | --- | --- | --- | --- | --- | --- | --- | --- | --- | --- |
|  |  |  |  |  |  |  | **Strong Sceptic** | | **Mild Sceptic** | | **Mild Trust** | | **Strong Trust** | |
|  |  |  | **Regression coefficient (log odds)** | **Cumulative odds ratio** | **Z value** | **p value** | **Mean** | **SE** | **Mean** | **SE** | **Mean** | **SE** | **Mean** | **SE** |
| Stewardship enablers:  *“Please rate your level of agreement with the following statements.”*  10-point scale (1=Very Strongly Disagree, 10=Very Strongly Agree) | I feel a sense of responsibility to help to improve waterway health | **Feel responsible** | **0.119** | **1.16** | **4.018** | **0.000** | **6.79** | 0.225 | **6.61** | 0.106 | **7.19** | 0.069 | **8.31** | 0.093 |
|  | I want to do more to help improve waterway health in my region | **Want to do more to help** | **0.119** | **1.15** | **4.270** | **0.000** | **6.21** | 0.236 | **6.56** | 0.104 | **7.02** | 0.066 | **8.06** | 0.096 |
|  | I can make a personal difference to improving waterway health in my region | **Personal efficacy** | **0.077** | **1.08** | **2.940** | **0.003** | **6.28** | 0.239 | **6.38** | 0.108 | **6.89** | 0.069 | **7.95** | 0.107 |
|  | Many local residents in my region are taking action to improve waterway health | Local residents support action | 0.046 | 1.05 | 1.622 | 0.105 | 5.35 | 0.234 | 5.60 | 0.095 | 6.21 | 0.064 | 6.48 | 0.106 |
|  | I feel hopeful about the future health of waterways in my region | Hopeful about the future | 0.044 | 1.05 | 1.931 | 0.054 | 5.91 | 0.255 | 6.16 | 0.106 | 6.59 | 0.068 | 6.99 | 0.113 |
|  | Local residents in my region are supportive of taking action to improve waterway health | Local residents taking action | 0.037 | 1.04 | 1.355 | 0.175 | 4.72 | 0.216 | 5.17 | 0.099 | 5.67 | 0.065 | 5.95 | 0.109 |
|  | I don’t have enough time to contribute to improving waterway health in my region | No time to contribute | 0.002 | 1.01 | 0.095 | 0.924 | 4.85 | 0.226 | 5.04 | 0.105 | 5.21 | 0.076 | 4.56 | 0.131 |
|  | I don’t know how I could contribute to improving waterway health in my region | Don't know how to contribute | -0.018 | 0.98 | -0.975 | 0.329 | 4.76 | 0.253 | 5.33 | 0.116 | 5.45 | 0.079 | 4.72 | 0.230 |
