# Supplementary material for: Finding common ground: Understanding and engaging with science mistrust in the Great barrier reef region
Source: PLoS One. 2024 Aug 16;19(8):e0308252. doi: 10.1371/journal.pone.0308252 (PMC11329155; doi:10.1371/journal.pone.0308252)
Supplement: S8 Table — (DOCX) [file pone.0308252.s008.docx]

**S8 Table.** **Results of ordinal regression models testing the relationship between survey respondents *’trust* [in] *the science about waterway health and management’* and predictor variables from survey questions about *perceptions of waterway health*, and mean rating scores (±SE) from four groups with differing stated *trust in science* (strongly sceptical, mildly sceptical, mildly trusting, strongly trusting) for each predictor variable**. Cumulative odds ratios indicate the predicted likelihood of increased or decreased *trust in science* corresponding to higher ratings in the predictor variable (values greater than one represent an increased likelihood while values less than one suggest decreased likelihoods). Variables with significant (p < 0.05) effects are indicated in bold font.

| Survey question and response options | Question items (waterway habitats) | Short variable name | Model results | | | | Mean rating scores (±SE) from four groups with differing stated trust in science | | | | | | | |
| --- | --- | --- | --- | --- | --- | --- | --- | --- | --- | --- | --- | --- | --- | --- |
|  |  |  |  |  |  |  | **Strong Sceptic** | | **Mild Sceptic** | | **Mild Trust** | | **Strong Trust** | |
|  |  |  | **Regression coefficient (log odds)** | **Cumulative odds ratio** | **Z value** | **p value** | **Mean** | **SE** | **Mean** | **SE** | **Mean** | **SE** | **Mean** | **SE** |
| Perceived health of waterway habitats:  *“Of the waterways you have visited in the region, how would you rate the health?”*  3-point scale (1 = In Poor Health, 2= In Fair Health, 3 = In Good Health) | Beaches & the coast | **Beaches & coast** | **0.364** | **1.44** | **2.641** | **0.008** | **2.49** | 0.056 | **2.47** | 0.030 | **2.55** | 0.020 | **2.54** | 0.028 |
|  | Freshwater, creeks & rivers | Freshwater | 0.164 | 1.18 | 1.146 | 0.252 | 2.28 | 0.062 | 2.38 | 0.031 | 2.46 | 0.021 | 2.39 | 0.032 |
|  | Estuaries | Estuaries | 0.082 | 1.09 | 0.591 | 0.554 | 2.38 | 0.067 | 2.41 | 0.030 | 2.43 | 0.021 | 2.37 | 0.030 |
|  | Offshore coral reefs | Offshore reefs | 0.009 | 1.01 | 0.068 | 0.960 | 2.54 | 0.066 | 2.26 | 0.037 | 2.19 | 0.026 | 2.10 | 0.036 |
|  | Lakes & dams | Lakes & dams | -0.090 | 0.91 | -0.628 | 0.530 | 2.39 | 0.059 | 2.38 | 0.032 | 2.46 | 0.021 | 2.43 | 0.031 |
|  | Seagrass | Seagrass | -0.192 | 0.82 | -1.607 | 0.108 | 2.16 | 0.069 | 2.12 | 0.032 | 2.07 | 0.025 | 1.92 | 0.035 |
|  | Inshore coral reefs | **Inshore reefs** | **-0.578** | **0.56** | **-4.444** | **0.000** | **2.37** | 0.071 | **2.01** | 0.039 | **1.92** | 0.026 | **1.78** | 0.036 |
|  | Ocean | Ocean | 0.100 | 1.10 | 0.681 | 0.496 | 2.52 | 0.058 | 2.35 | 0.033 | 2.38 | 0.023 | 2.36 | 0.031 |
