# Supplementary material for: Finding common ground: Understanding and engaging with science mistrust in the Great barrier reef region
Source: PLoS One. 2024 Aug 16;19(8):e0308252. doi: 10.1371/journal.pone.0308252 (PMC11329155; doi:10.1371/journal.pone.0308252)
Supplement: S9 Table — (DOCX) [file pone.0308252.s009.docx]

**S9 Table.** **Results of ordinal regression models testing the relationship between survey respondents *’trust* [in] *the science about waterway health and management’* and predictor variables from survey questions about *perceptions of waterway problems*, and mean rating scores (±SE) from four groups with differing stated *trust in science* (strongly sceptical, mildly sceptical, mildly trusting, strongly trusting) for each predictor variable**. Cumulative odds ratios indicate the predicted likelihood of increased or decreased *trust in science* corresponding to higher ratings in the predictor variable (values greater than one represent an increased likelihood while values less than one suggest decreased likelihoods). Variables with significant (p < 0.05) effects are indicated in bold font.

| Survey question and response options | Question items | Short variable name | Model results | | | | Mean rating scores (±SE) from four groups with differing stated trust in science | | | | | | | |
| --- | --- | --- | --- | --- | --- | --- | --- | --- | --- | --- | --- | --- | --- | --- |
|  |  |  |  |  |  |  | **Strong Sceptic** | | **Mild Sceptic** | | **Mild Trust** | | **Strong Trust** | |
|  |  |  | **Regression coefficient**  **(log odds)** | **Cumulative odds ratio** | **Z value** | **p value** | **Mean** | **SE** | **Mean** | **SE** | **Mean** | **SE** | **Mean** | **SE** |
| Perceived problems in regional waterways:  *“From your experience, how problematic do you think each of the following issues are for waterways in the region?”*  5-point scale (1 = Not a problem at all, 2 = A small problem, 3 = A moderate problem, 4 = A big problem, 5 = A very big problem) | Chemical pollutants (e.g. pesticides, PFAS) | **Chemical pollutants** | **0.210** | **1.23** | **3.123** | **0.002** | **3.040** | 0.114 | **3.380** | 0.052 | **3.34** | 0.036 | **3.55** | 0.056 |
|  | Riverbank erosion | **Riverbank erosion** | **0.142** | **1.15** | **2.029** | **0.042** | **3.10** | 0.130 | **3.56** | 0.060 | **3.74** | 0.038 | **3.82** | 0.055 |
|  | Coastal erosion | Coastal erosion | 0.091 | 1.10 | 1.375 | 0.169 | 2.80 | 0.124 | 3.26 | 0.056 | 3.38 | 0.036 | 3.49 | 0.056 |
|  | Poor offshore marine water quality | Offshore water quality | 0.067 | 1.07 | 0.992 | 0.321 | 2.66 | 0.129 | 3.02 | 0.062 | 3.20 | 0.042 | 3.25 | 0.063 |
|  | Poor coastal and inshore water quality | Coastal water quality | 0.062 | 1.06 | 0.805 | 0.421 | 2.88 | 0.125 | 3.18 | 0.061 | 3.32 | 0.039 | 3.46 | 0.060 |
|  | Weeds in and around waterways | Weeds in waterways | 0.015 | 1.02 | 0.246 | 0.806 | 3.52 | 0.115 | 3.62 | 0.053 | 3.61 | 0.036 | 3.74 | 0.053 |
|  | Poor water quality in fresh waters and estuaries | Freshwater water quality | -0.007 | 0.99 | -0.100 | 0.920 | 2.93 | 0.124 | 3.29 | 0.057 | 3.33 | 0.040 | 3.49 | 0.060 |
|  | Algal blooms & fish kills | Algal blooms & fish kills | -0.033 | 0.98 | -0.535 | 0.593 | 3.06 | 0.124 | 3.40 | 0.058 | 3.46 | 0.040 | 3.56 | 0.056 |
|  | Invasive fish (e.g. Tilapia) | Invasive fish | -0.083 | 0.92 | -1.401 | 0.161 | 3.74 | 0.106 | 3.72 | 0.051 | 3.78 | 0.036 | 3.82 | 0.051 |
|  | Litter and debris (e.g. plastics) | Litter & debris | -0.102 | -0.90 | -1.518 | 0.129 | 3.83 | 0.104 | 3.97 | 0.050 | 3.98 | 0.035 | 4.05 | 0.048 |
|  | Low abundance of fish | Low fish abundance | -0.114 | 0.89 | -1.879 | 0.060 | 3.29 | 0.119 | 3.46 | 0.057 | 3.42 | 0.039 | 3.55 | 0.056 |
