# Supplementary material for: Finding common ground: Understanding and engaging with science mistrust in the Great barrier reef region
Source: PLoS One. 2024 Aug 16;19(8):e0308252. doi: 10.1371/journal.pone.0308252 (PMC11329155; doi:10.1371/journal.pone.0308252)
Supplement: S10 Table — (DOCX) [file pone.0308252.s010.docx]

**S10 Table.** **Results of ordinal regression models testing the relationship between survey respondents *’trust* [in] *the science about waterway health and management’* and predictor variables from survey questions about *perceptions of threats to regional waterways*, and mean rating scores (±SE) from four groups with differing stated *trust in science* (strongly sceptical, mildly sceptical, mildly trusting, strongly trusting) for each predictor variable**. Cumulative odds ratios indicate the predicted likelihood of increased or decreased *trust in science* corresponding to higher ratings in the predictor variable (values greater than one represent an increased likelihood while values less than one suggest decreased likelihoods). Variables with significant (p < 0.05) effects are indicated in bold font.

| Survey question and response options | Question items | Short variable name | Model results | | | | Mean rating scores (±SE) from four groups with differing stated trust in science | | | | | | | |
| --- | --- | --- | --- | --- | --- | --- | --- | --- | --- | --- | --- | --- | --- | --- |
|  |  |  |  |  |  |  | **Strong Sceptic** | | **Mild Sceptic** | | **Mild Trust** | | **Strong Trust** | |
|  |  |  | **Regression coefficient** | **Cumulative odds ratio** | **Z value** | **p value** | **Mean** | **SE** | **Mean** | **SE** | **Mean** | **SE** | **Mean** | **SE** |
| Perceived threats:  *“For the following list of issues – please rate the extent to which you think they represent a current threat to waterways in the region.”*  5-point scale (1 = Does not represent a threat at all, 2 = A minor threat, 3 = A moderate threat, 4 = A serious threat, 5 = Represents an extremely serious threat) | Climate change | **Climate change** | **0.652** | **1.92** | **12.269** | **0.000** | **2.23** | 0.121 | **3.05** | 0.066 | **3.53** | 0.042 | **4.00** | 0.054 |
|  | Land clearing | Land clearing | 0.109 | 1.11 | 1.762 | 0.078 | 3.01 | 0.125 | 3.38 | 0.058 | 3.50 | 0.366 | 3.79 | 0.052 |
|  | Sediments, nutrients and pesticides from land-based runoff | Land-based runoff | 0.113 | 1.12 | 1.754 | 0.079 | 2.96 | 0.122 | 3.43 | 0.056 | 3.56 | 0.034 | 3.85 | 0.049 |
|  | Recreation activities (e.g. boats, fishers, water sports) | Recreation activities | 0.100 | 1.11 | 1.525 | 0.127 | 2.24 | 0.100 | 2.60 | 0.062 | 2.58 | 0.035 | 2.59 | 0.046 |
|  | Extreme weather (e.g. cyclones, droughts, floods) | Extreme weather | 0.095 | 1.10 | 1.732 | 0.083 | 2.60 | 0.111 | 3.17 | 0.050 | 3.36 | 0.034 | 3.56 | 0.049 |
|  | Illegal fishing practices (e.g. poaching in no-take zones) | Illegal fishing practices | -0.008 | 0.99 | -0.150 | 0.880 | 3.54 | 0.097 | 3.59 | 0.051 | 3.62 | 0.036 | 3.72 | 0.048 |
|  | Over-fishing | Over-fishing | -0.053 | 0.95 | -0.857 | 0.391 | 3.33 | 0.111 | 3.50 | 0.052 | 3.52 | 0.034 | 3.70 | 0.049 |
|  | Mining activities (e.g. water extraction, releases) | Mining | -0.060 | 0.94 | -1.040 | 0.297 | 2.94 | 0.123 | 3.34 | 0.059 | 3.49 | 0.038 | 3.65 | 0.057 |
|  | Tourism activities | **Tourism activities** | **-0.306** | **0.74** | **-4.522** | **0.000** | **2.56** | 0.101 | **2.56** | 0.048 | **2.57** | 0.028 | **2.59** | 0.044 |
