# Supplementary material for: Finding common ground: Understanding and engaging with science mistrust in the Great barrier reef region
Source: PLoS One. 2024 Aug 16;19(8):e0308252. doi: 10.1371/journal.pone.0308252 (PMC11329155; doi:10.1371/journal.pone.0308252)
Supplement: Attachment — Submitted filename: Response to Reviewers.docx [file pone.0308252.s011.docx]

**PONE-D-24-08298	PLOS ONE	17 July 2024**

*Finding Common Ground: understanding and engaging with science mistrust in the Great Barrier Reef region*

**Response to Reviewers and Editor:**

On behalf of my co-authors, thank you for your consideration and careful review of our manuscript ‘*Finding Common Ground: understanding and engaging with science mistrust in the Great Barrier Reef region’*. We are grateful for your constructive and positive feedback, and we have done our best to address the reviewers’ feedback, as outlined in Table 1 below. Responses to editorial feedback on the journal’s requirements are provided in Table 2. We appreciate the opportunity to revise and resubmit our manuscript, which we hope is now worthy of publication in PLoS ONE.

Kind regards,

Matt Curnock (*corresponding author*)

**Table 1: Response to reviewers**

| **Reviewer feedback** | **Author response** |
| --- | --- |
| **Reviewer #1:**  This manuscript is both comprehensive and thorough its content. Having read through and continually had comments I had written addressed by the next page or section read, I have no comments to submit beyond stating that I think this paper should be accepted. The work is insightful, meets the scope of PLOS ONE and I think would be valued by the journals readership. | We are very grateful for this positive feedback. Thank you! |
| **Reviewer #2:**  This manuscript describes the results of a large public survey in Australia in the region near the Great Barrier Reef. In particular findings explore public trust in scientific natural resource management as it relates to other attitudes, values, and individual differences. Overall, I found the paper interesting and well written. The methods were appropriate, sound, and well described.  My only note is that some further treatment of how the key variables of values, attitudes toward civic institutions, and trust in science may be related in this region to partisan political identity. The authors touch on these subjects in the intro and discussion. Particularly given the association with some demographic data (gender, industry of employment), the importance of one's identifying with the values and attitudes of one's social group feels like it deserves further consideration here. In the US at least, trust in the realm of policy has become increasingly partisan, making traditional communication strategies to overcome mistrust more complicated to say the least. Even just a bit more coverage of the political context in which management and communication is taking place may be helpful. You touch on some of this in lines ~288-297 for instance, but it left me wanting to know a bit more. For instance, what are the major sources of misinformation in this region specifically and what interests are they bringing to the table.  I'll emphasize, that I don't want to make it seem like I am asking the authors to write a different paper with different questions/aims/variables. I'd just like to to see a bit more concrete grounding for the context and discuss the role of that context in interpreting results and implications. | Thank you!  We are aware that political identity plays an important role in people’s attitudes towards civic institutions and their trust in science in Australia, as it does in the USA and other countries. While it was beyond the scope of our study to investigate the relative influence of political identity and other potential drivers of mistrust in science, we agree that this is important context. In our manuscript (version with track changes) we have referred to the broader political context and its relationship to trust in science on lines 52-54, 692-694, and 734-735. We make note of the role of politics in the context of our GBR case study on lines 273-278, and we have added some additional text in the Discussion section (lines 702-705), expanding on key findings by Lubicz-Zaorski et al. (2023), whose study represents the only empirical account of a politically motivated misinformation campaign in the GBR to date:  “*An empirical assessment of social media posts by Lubicz-Zaorski et al. [98] found that a small group of politically aligned actors were responsible for a campaign of misinformation specifically about the GBR, with an apparent motive to erode public support for Australian climate change policy*.”  Please note that the authors are cognizant of a need to frame this manuscript in an objective and apolitical manner, to avoid any perceptions of political bias on our part. We are reluctant to expand on this issue further without an empirical basis relevant to our regional case study. |

**Table 2: Response to editor(s)**

| **Editorial feedback (journal requirements)** | **Author response** |
| --- | --- |
| 1. Please ensure that your manuscript meets PLOS ONE's style requirements, including those for file naming. | We have updated the manuscript in accordance with PLOS ONE’s style requirements, including the referencing style and titles for display items. File names have also been updated as required. |
| 2. Please state what role the funders took in the study. If the funders had no role, please state: ""The funders had no role in study design, data collection and analysis, decision to publish, or preparation of the manuscript.""  If this statement is not correct you must amend it as needed.  Please include this amended Role of Funder statement in your cover letter; we will change the online submission form on your behalf. | We confirm that the funders had no role in the study design, data collection and analysis, decision to publish, or preparation of the manuscript.  An updated financial disclosure/funding statement is provided below this table. |
| 3. We note that you have provided funding information that is not currently declared in your Funding Statement. However, funding information should not appear in the Acknowledgments section or other areas of your manuscript. We will only publish funding information present in the Funding Statement section of the online submission form.  Please remove any funding-related text from the manuscript and let us know how you would like to update your Funding Statement. | We have removed the funding information from Acknowledgements section in the manuscript.  The Acknowledgements section in the manuscript (after the Conclusion section) now states:  “This research was conducted using data from the Social and Economic Long-Term Monitoring Program for the Great Barrier Reef (SELTMP; <https://research.csiro.au/seltmp>), collected in partnership with the Queensland Office of the Great Barrier Reef and World Heritage, and the five Regional Report Card Partnerships in the Great Barrier Reef catchment region: Wet Tropics Waterways, Dry Tropics Partnership for Healthy Waters, Mackay-Whitsunday-Isaac Healthy Rivers to Reef Partnership, Fitzroy Partnership for River Health, and Gladstone Healthy Harbour Partnership. The scientific results and conclusions, as well as any views or opinions expressed herein, are those of the authors and do not necessarily reflect the views of the Australian Government or the Queensland Government and their respective Ministers for the Environment.”  An amended funding statement is provided below this table. |
| 4. Please include captions for your Supporting Information files at the end of your manuscript, and update any in-text citations to match accordingly. Please see our Supporting Information guidelines for more information: <http://journals.plos.org/plosone/s/supporting-information> | Captions for Supporting Information Tables (S1 to S10) are now provided at the end of the manuscript, above the Author Contributions and References. |
| 5. Please review your reference list to ensure that it is complete and correct. If you have cited papers that have been retracted, please include the rationale for doing so in the manuscript text, or remove these references and replace them with relevant current references. Any changes to the reference list should be mentioned in the rebuttal letter that accompanies your revised manuscript. If you need to cite a retracted article, indicate the article’s retracted status in the References list and also include a citation and full reference for the retraction notice. | We have reviewed our reference list, updated the style to PLOS ONE requirements, and we are confident that it is complete and correct.  Note that we have added one additional citation to a newly published report (Hobman et al., 2024; #107) which provides current findings on GBR residents’ declining trust in science. We had previously referred to this in the Discussion section as Hobman et al. in review.  The manuscript does not contain any citations to papers that have been retracted. |
| **Additional Editor Comments:**  This manuscript has received glowing reviews, and it will provide a valuable contribution to the literature and to our journal's readership.  Please consider the feedback provided by Reviewer #2. I would suggest the authors to consider providing a bit of information on the Australian political context in relation to the Australian Great Barrier Reef.  Thank you for choosing PLOS One for publishing this high-quality piece of research. | We are very grateful for this positive feedback, and we are hopeful that the findings from the study will be useful to scientists, practitioners and resource managers in the Great Barrier Reef region and elsewhere.  We are appreciative of Reviewer 2’s suggested additions and have done our best to address this point, as described in the table above, acknowledging that it was beyond our study’s scope to identify specific drivers of science mistrust in the GBR region. We note that we have endeavoured to ensure our study’s findings and implications are grounded in evidence relevant to the case study region. We also wish to avoid any potential perceptions of political bias on the authors’ part.  Thank you! |

**Financial disclosure/funding statement (amended):**

Funding for this study was provided by the partnership between the Australian Government’s Reef Trust and the Great Barrier Reef Foundation, delivered in partnership with CSIRO, the Great Barrier Reef Marine Park Authority, and the Queensland Government’s Reef Water Quality Program. The funders had no role in study design, data collection and analysis, decision to publish, or preparation of the manuscript.
